# Assessing Granger Causality in Electrophysiological Data: Removing the Adverse Effects of Common Signals via Bipolar Derivations

**DOI:** 10.3389/fnsys.2015.00189

**Published:** 2016-01-20

**Authors:** Amy Trongnetrpunya, Bijurika Nandi, Daesung Kang, Bernat Kocsis, Charles E. Schroeder, Mingzhou Ding

**Affiliations:** ^1^J. Crayton Pruitt Family Department of Biomedical Engineering, University of FloridaGainesville, FL, USA; ^2^Department of Psychiatry at Beth Israel Deaconess Medical Center, Harvard Medical SchoolBoston, MA, USA; ^3^Nathan S. Kline Institute for Psychiatric ResearchOrangeburg, NY, USA; ^4^Department of Neurosurgery, Columbia UniversityNew York, NY, USA

**Keywords:** bipolar signals, unipolar signals, Granger causality, hippocampus, V4, ECoG

## Abstract

Multielectrode voltage data are usually recorded against a common reference. Such data are frequently used without further treatment to assess patterns of functional connectivity between neuronal populations and between brain areas. It is important to note from the outset that such an approach is valid only when the reference electrode is nearly electrically silent. In practice, however, the reference electrode is generally not electrically silent, thereby adding a common signal to the recorded data. Volume conduction further complicates the problem. In this study we demonstrate the adverse effects of common signals on the estimation of Granger causality, which is a statistical measure used to infer synaptic transmission and information flow in neural circuits from multielectrode data. We further test the hypothesis that the problem can be overcome by utilizing bipolar derivations where the difference between two nearby electrodes is taken and treated as a representation of local neural activity. Simulated data generated by a neuronal network model where the connectivity pattern is known were considered first. This was followed by analyzing data from three experimental preparations where *a priori* predictions regarding the patterns of causal interactions can be made: (1) laminar recordings from the hippocampus of an anesthetized rat during theta rhythm, (2) laminar recordings from V4 of an awake-behaving macaque monkey during alpha rhythm, and (3) ECoG recordings from electrode arrays implanted in the middle temporal lobe and prefrontal cortex of an epilepsy patient during fixation. For both simulation and experimental analysis the results show that bipolar derivations yield the expected connectivity patterns whereas the untreated data (referred to as unipolar signals) do not. In addition, current source density signals, where applicable, yield results that are close to the expected connectivity patterns, whereas the commonly practiced average re-reference method leads to erroneous results.

## Introduction

Multielectrode voltage data, recorded typically with respect to a common reference electrode, form the foundation for inferring interaction patterns among neuronal ensembles in the brain. If the electrical activity at the common reference electrode is not negligible then it becomes a common signal present at all electrodes. Another source of common signal is volume conduction (Nunez et al., 1997; Mima and Hallett, 1999; Kajikawa and Schroeder, 2011, 2015; Tenke and Kayser, 2012). Recognizing and overcoming issues associated with the common signal problem is essential for the proper application and interpretation of functional connectivity measures derived from multielectrode data.

To illustrate the common signal problem, consider the impact of common reference. Let *X*_1_(*t*) and *X*_2_(*t*) denote neural activities at two recording sites. Let *R*(*t*) denote electrical activity at the reference electrode. It is important to realize that the readings from the two recording electrodes are *X*_1_(*t*) − *R*(*t*) and *X*_2_(*t*) − *R*(*t*) rather than *X*_1_(*t*) and *X*_2_(*t*). [Here we refer to *X*_1_(*t*) − *R*(*t*) and *X*_2_(*t*) − *R*(*t*) as unipolar signals in contrast to the bipolar signals to be defined below]. If, under ideal conditions, the magnitude of the common signal *R*(*t*) is nearly zero or negligible in comparison to *X*_1_(*t*) and *X*_2_(*t*), statistical connectivity inferred from *X*_1_(*t*) − *R*(*t*) and *X*_2_(*t*) − *R*(*t*) can be equated to that inferred from *X*_1_(*t*) and *X*_2_(*t*), which in turn reflects the functional interaction between the neuronal ensembles at the two recording sites. In practice, however, this is generally not the case. The magnitude of *R*(*t*) is not negligible in many experimental settings, and in fact, it can be as large as the neural activities *X*_1_(*t*) and *X*_2_(*t*) themselves. Our first goal is to examine the adverse effects of common signals on the estimation of Granger causality, a measure of synaptic transmission and directional information flow in neuronal circuits (Ding et al., 2006).

A solution to the common signal problem may lie inherently in the multielectrode recording itself. It is quite common that multiple electrodes are placed in the same brain structure or neuronal ensemble. Examples include tetrodes and multicontact laminar electrodes (Schroeder et al., 1998; Lakatos et al., 2008; Hansen et al., 2012; Leopold and Maier, 2012; Kapoor et al., 2013; Newman et al., 2013; Godlove et al., 2014). Consider two brain areas. Suppose that *X*_1_(*t*) and *Y*_1_(*t*) denote neural activities under two electrodes in Area 1 and *X*_2_(*t*) and *Y*_2_(*t*) denote neural activities under two electrodes in Area 2. Data from the four electrodes are *X*_1_(*t*) − *R*(*t*), *Y*_1_(*t*) − *R*(*t*), *X*_2_(*t*) − *R*(*t*), and *Y*_2_(*t*) − *R*(*t*), respectively. The common signal *R*(*t*) can be removed by taking a bipolar derivation within Area 1: [*X*_1_(*t*) − *R*(*t*)] − [*Y*_1_(*t*) − *R*(*t*)] = *X*_1_(*t*) − *Y*_1_(*t*) and within Area 2: [*X*_2_(*t*) − *R*(*t*)] − [*Y*_2_(*t*) − *R*(*t*)] = *X*_2_(*t*) − *Y*_2_(*t*). After such bipolar treatment *X*_1_(*t*) − *Y*_1_(*t*) and *X*_2_(*t*) − *Y*_2_(*t*) are free of the common signal, and represent local neuronal activity in Area 1 and Area 2. Volume conduction can be removed this way as well if the two electrodes in each of the two areas are sufficiently close. Using bipolar derivations to mitigate the effects of common reference and volume conduction has been considered in human scalp EEG by Nunez et al. (1997, 1999). However, in invasive recordings, because electrodes are closer to the recorded tissue, this problem has traditionally not been given sufficient attention. Yet, the same theoretical principle illustrated above applies to invasive as well as non-invasive recordings. Earlier work using laminar recordings from monkey visual cortex demonstrates that erroneous estimates of Granger causality may arise from unipolar data and the use of bipolar derivations yielded the expected results (Bollimunta et al., 2009). Our second goal is thus to demonstrate that bipolar derivation is an effective method to overcome the common signal problem.

Four datasets were analyzed to achieve our goals, including: (1) simulated data from a neuronal model of two coupled brain areas where the exact pattern of connectivity is known, (2) laminar recordings from the hippocampus of an anesthetized rat during theta rhythm, (3) laminar recordings from V4 of an awake-behaving monkey during alpha rhythm, and (4) intracranial ECoG recordings from a human epilepsy patient during fixation. In each case, *a priori* predictions can be made on the directionality of synaptic transmission and information flow, thereby furnishing the ground truth upon which the performance of bipolar data and other treatments of data including unipolar data, average re-referenced data and current source density data is evaluated.

## Methods

### Sources of data

#### Simulation model

**Figure 1:**
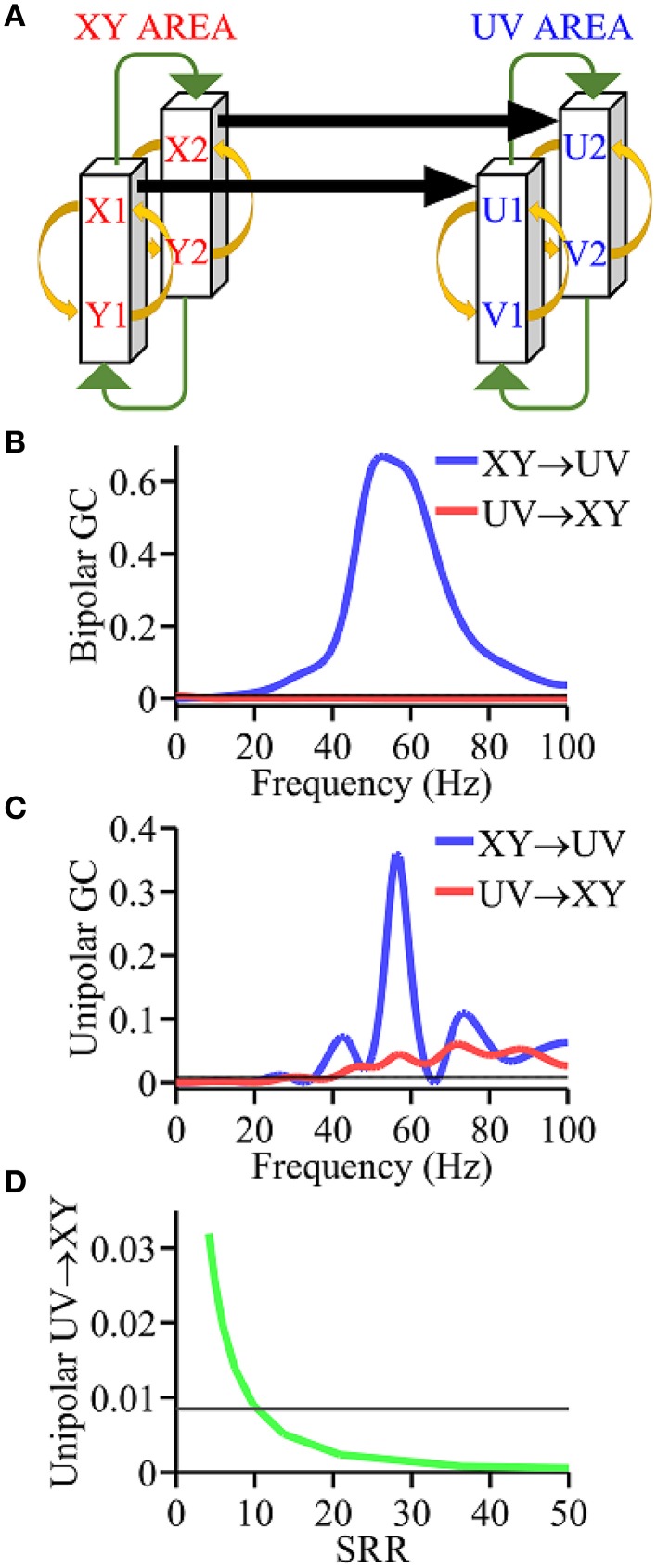
**Simulation model. (A)** Coupling scheme. **(B)** Granger causality spectra using bipolar signals which are in agreement with ground truth. **(C)** Granger causality spectra using unipolar signals which are not in agreement with ground truth. **(D)** Analysis of the unipolar Granger causality UV → XY as a function of the signal to reference ratio (SRR) (green curve). Black horizontal lines in **(B–D)** represents the level of statistical significance (*p* < 0.05).

The model had two interacting brain areas, XY area and UV area, with each comprised of two coupled cortical columns where each column was made up of an excitatory and an inhibitory neuronal population (Kamiński et al., 2001). A schematic of the model is given in Figure 1A. The equations governing the dynamics of the XY area are given by:

$$\begin{array}{l} \frac{d^{2}x_{1}}{dt^{2}}+\left(a+b\right)\frac{dx_{1}}{dt}+abx_{1}=-k_{ie}Q\left(y_{1}\left(t\right),Q_{m0}\right)\\ \;+\;k_{12}Q\left(x_{1}\left(t\right),Q_{m0}\right)+\xi_{x\text{1}}(t)\\ \frac{d^{2}y_{1}}{dt^{2}}+\left(a+b\right)\frac{dy_{1}}{dt}+aby_{1}=k_{ei}Q\left(x_{1}\left(t\right),Q_{m0}\right)+\xi_{y\text{1}}(t)\\ \frac{d^{2}x_{2}}{dt^{2}}+\left(a+b\right)\frac{dx_{2}}{dt}+abx_{2}=-k_{ie}Q\left(y_{2}\left(t\right),Q_{m0}\right)\\ \;+\;k_{12}Q\left(x_{2}\left(t\right),Q_{m0}\right)+\xi_{x\text{2}}(t)\\ \frac{d^{2}y_{2}}{dt^{2}}+\left(a+b\right)\frac{dy_{2}}{dt}+aby_{2}=k_{ei}Q\left(x_{2}\left(t\right),Q_{m0}\right)+\xi_{y\text{2}}(t) \end{array}$$

and the equations governing the dynamics of the UV area are given by:

$$\begin{array}{l} \frac{d^{2}u_{1}}{dt^{2}}+\left(a+b\right)\frac{du_{1}}{dt}+abu_{1}=-k_{ie}Q\left(v_{1}\left(t\right),Q_{m0}\right)\\ \;+\;k_{21}Q\left(u_{1}\left(t\right),Q_{m0}\right)\\ \;+\;k_{\text{XU}}Q\left(x_{1}\left(t\right),Q_{m0}\right)+\xi_{u\text{1}}(t)\\ \frac{d^{2}v_{1}}{dt^{2}}+\left(a+b\right)\frac{dv_{1}}{dt}+abv_{1}=k_{ei}Q\left(u_{1}\left(t\right),Q_{m0}\right)+\xi_{v\text{1}}(t) \end{array}$$

$$\begin{array}{l} \frac{d^{2}u_{2}}{dt^{2}}+\left(a+b\right)\frac{du_{2}}{dt}+abu_{2}=-k_{ie}Q\left(v_{2}\left(t\right),Q_{m0}\right)\\ \;+\;k_{21}Q\left(u_{2}\left(t\right),Q_{m0}\right)\\ \;+\;k_{XU}Q\left(x_{2}\left(t\right),Q_{m0}\right)+\xi_{u\text{2}}(t)\\ \frac{d^{2}v_{2}}{dt^{2}}+\left(a+b\right)\frac{dv_{2}}{dt}+abv_{2}=k_{ei}Q\left(u_{2}\left(t\right),Q_{m0}\right)+\xi_{v\text{2}}(t) \end{array}$$

Here *x*(*t*), *u*(*t*), and *y*(*t*), *v*(*t*) represent local field potentials (LFP) of the excitatory and inhibitory populations, respectively, *k*_*ei*_ > 0 gives the coupling gain from the excitatory (*X, U*) to the inhibitory(*Y, V*) population, and *k*_ie_ > 0 is the strength of the reciprocal coupling. Between XY area and UV area, the model is constructed in such a way that there is unidirectional driving from XY area to UV area with a non-zero coupling strength *k*_XU_, resulting in XY → UV > 0, and the driving in the opposite direction is zero, UV → XY = 0. This is the ground truth for the connectivity pattern in the simulation model. Neuronal coupling in the model is assumed to be mediated through a sigmoidal function *Q*(*g, Q*_*mo*_), which represents the pulse densities converted from g with *Q*_*mo*_ as a modulatory parameter (Freeman, 1992), and is defined by,

$$\begin{array}{l} \;Q\left(g,Q_{m0}\right)=Q_{m0}[\text{1}-{\text{e}}^{-({\text{e}}^{\text{g}}-\text{1}){\text{/Q}}_{\text{m0}}}]\text{ if }g>\;-\;h_{0}\\ \text{or }Q\left(g,\;Q_{m0}\right)=-\text{1 if }g<\;-\;h_{0} \end{array}$$

where *h*_0_ = −ln[1 + ln(1 + 1 ∕ *Q*_*m*0_)]. The term ξ(*t*) in each equation represents independent Gaussian white noise input.

The above system of differential equations were solved using a fourth order Runge-Kutta method with a time step of 0.1 ms. The simulated dataset was 100 s in duration, sampled at 200 Hz, and divided into 500 ms epochs. The parameter values used were: *a* = 0.22∕*ms*, *b* = 0.72∕*ms*, *k*_XU_ = 0.25, *k*_12_ = 0.001, *k*_21_ = 0.001, *k*_*ei*_ = 0.1, *k*_*ie*_ = 2.5, *Q*_*m*0_ = 5. The standard deviation σ_ξ_ of input white noise process is chosen as 0.2. The common reference signal *R*(*t*) is also a Gaussian white noise process with a standard deviation of 0.2 for the main simulation. The unipolar signals are *x*_1_(*t*) − *R*(*t*), *x*_2_(*t*) − *R*(*t*), *u*_1_(*t*) − *R*(*t*) and *u*_2_(*t*) − *R*(*t*), and the bipolar signals representing the XY area and the UV area are *x*_1_(*t*) − *x*_2_(*t*) and *u*_1_(*t*) − *u*_2_(*t*), respectively. The signal to reference ratio (SRR) was calculated as the ratio between the amplitude of *x*_1_(*t*) and *R*(*t*). SRR is controlled by changing the standard deviation of *R*(*t*).

#### Laminar recordings from rat hippocampus

**Figure 2:**
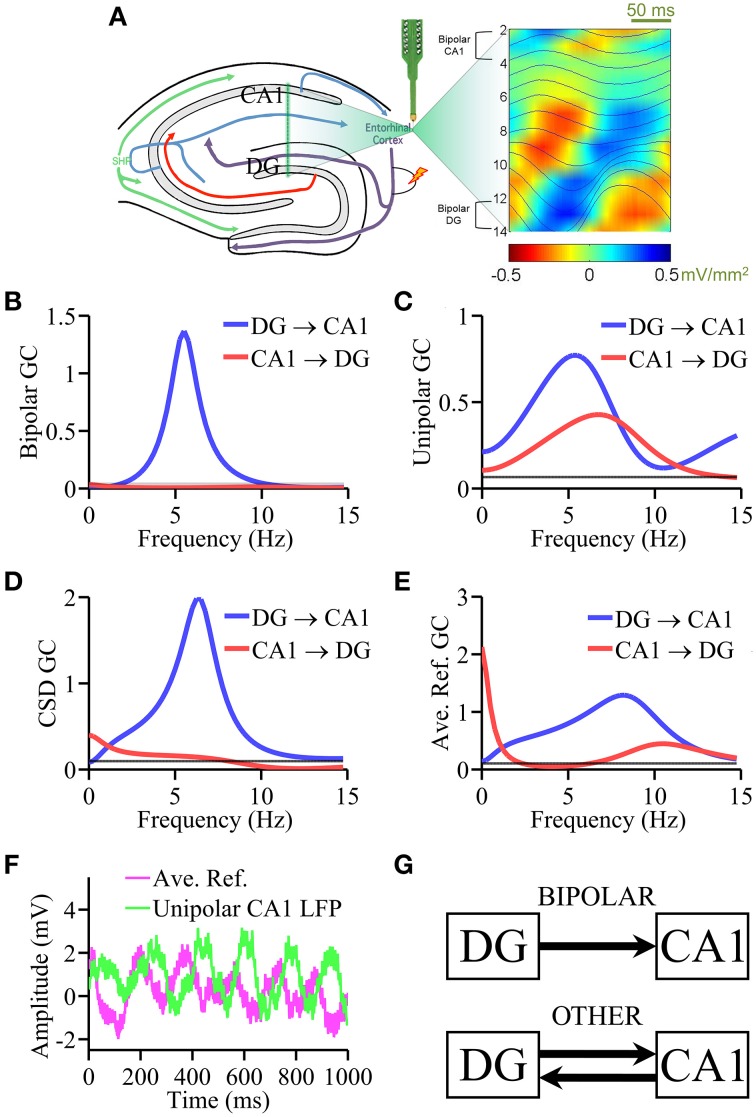
**Laminar recording from rat hippocampus**. **(A)** Schematic of the multielectrode with 16 equally spaced (100 μm) contacts and theta generators as identified by phase realigned and averaged (PRAT) CSD (color-coded) and LFPs (smooth traces, blue). Granger causality spectral analysis using **(B)** bipolar data, **(C)** unipolar data, **(D)** CSD data, and **(E)** average re-referenced data. **(F)** Comparison of unipolar LFPs from CA1 and average reference signals. **(G)** Schematic summarizing the Granger causality results where “OTHER” includes unipolar data, average re-referenced data and CSD data. Bipolar results are in agreement with ground truth. Black horizontal lines in **(B–E)** represents the level of statistical significance (*p* < 0.05).

All surgical and other relevant aspects of the experimental procedure were approved by the Institutional Animal Care and Use Committee. Hippocampal local field potentials (LFPs) were recorded from a rat under urethane anesthesia using a 16-channel linear multicontact electrode with 100 μm separation between contacts (Channel 16 was not used for recording LFPs). The linear electrode (NeuroNexus A1x10, 50 μm in diameter) was implanted in the dorso-ventral direction to cover a 1.5–2.5 mm segment across CA1, DG, and hilar regions as illustrated in Figure 2A. Two stainless steel watch screws, driven into the bone above the cerebellum and digitally averaged, served as reference electrodes (Kocsis et al., 1999). LFP was recorded using A-M System Model-3600 amplifiers set at a gain of 5000, sampled at 10 kHz with 12-bit precision, low-pass filtered offline (<250 Hz), and downsampled to 200 Hz. Data was divided into epochs of 2 s in duration. A total of 49 epochs were analyzed here. Contact impedances were 1.7–2.5 MΩ at 1000 Hz. CA1 and DG regions were identified by perforant path evoked potentials. Theta rhythm was elicited by high frequency (100 Hz) stimulation of the pontine reticular formation (Kocsis et al., 1999). Theta generators were identified using the PRAT CSD method (Bollimunta et al., 2008, 2011), where (1) the power spectrum for each channel was estimated using FFT with 50% overlapping moving windows, and the channel showing the highest power spectral density at theta frequency was chosen as the “phase index contact;” (2) a sinusoid of theta peak frequency was then fitted to the phase index contact for each epoch; (3) the LFP data from all the channels of one epoch were shifted according to the estimated phase such that phase relations were conserved; (4) shifted and realigned signals were averaged across epochs; (5) a second spatial derivative was calculated to produce the CSD profile (Schroeder et al., 1995), from which the theta generators were identified. A similar method was used to study the gamma oscillatory activity in the hippocampus (Csicsvari et al., 2003).

Anatomically proven pathways exist within the hippocampus to carry synaptic transmission from DG to CA1 through the classic three-synaptic network, namely, DG → CA3 → CA1 via mossy fibers and Schaffer collaterals (Andersen et al., 1979; Amaral and Witter, 1989; Amaral et al., 2007); neurons projecting from the CA1 to DG are extremely sparse. This provided the “ground truth” for testing various types of data. Unipolar LFPs were taken from the channels overlaying the theta dipoles in the DG hilus region (contact 13) and in CA1 (contact 3). For bipolar LFPs, the contacts used for the hippocampal bipolar derivations were: CA1 = LFP(contact 2) − LFP(contact 4) and DG = LFP(contact 12) − LFP(contact 14), as shown in Figure 2A. For average re-referenced signals the average across all recording channels (contacts 1–15) was computed and subtracted from each channel. The second derivative approximation was applied to LFPs in each epoch to generate single-trial current source density (CSD) signals (Mitzdorf, 1985). The ground truth prediction is that DG → CA1 is expected to be large and significant whereas CA1 → DG small and insignificant.

#### Laminar recordings from monkey V4

**Figure 3:**
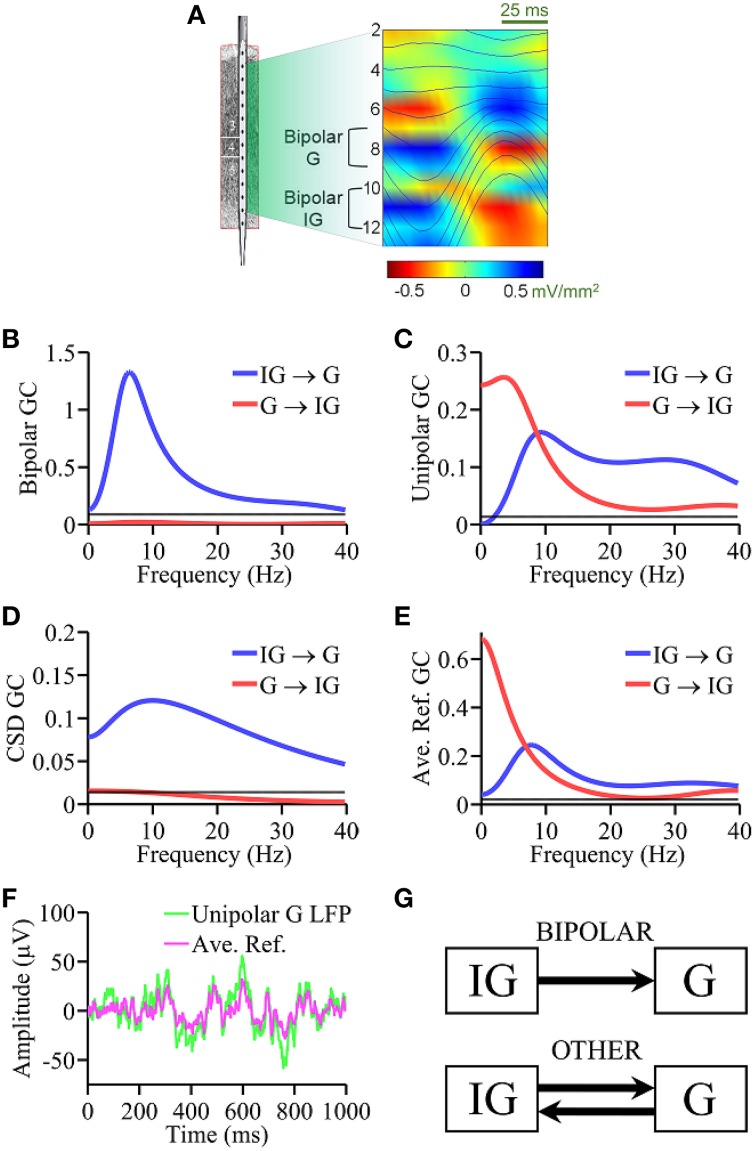
**Laminar recording from monkey V4. (A)** Schematic of the multielectrode with 14 equally spaced (200 μm) contacts and alpha generators as identified by phase realigned and averaged (PRAT) CSD (color-coded) and LFPs (smooth traces, blue). Granger causality spectral analysis using **(B)** bipolar data, **(C)** unipolar data, **(D)** CSD data, and **(E)** average re-referenced data. **(F)** Comparison of unipolar LFPs from granular layer and average reference signals. **(G)** Schematic summarizing the Granger causality results where “OTHER” includes unipolar data, average re-referenced data and CSD data. Bipolar results are in agreement with ground truth. Black horizontal lines in **(B–E)** represents the level of statistical significance (*p* < 0.05).

All surgical, training, and other relevant aspects of the experimental procedure were approved by the Institutional Animal Care and Use Committee. LFPs were recorded at 2 kHz using a multi-contact linear electrode with 14 recording contacts spanning all six cortical layers in visual area V4 of a macaque monkey performing auditory stimulus discrimination (Schroeder et al., 1998; Mehta et al., 2000; Chen et al., 2007; see Figure 3A). The separation between contacts was 200 μm. Contact impedances were 0.3 MΩ at 1000 Hz. The epidural guide tubes positioned over central and frontal sites served as reference electrodes (Bollimunta et al., 2008). The data was recorded using Grass P5 amplifiers set at a gain of 5000 and a bandpass of 3 Hz–3 kHz. The data was downsampled to 200 Hz and further divided into epochs of 200 ms in duration. A total of 201 epochs were analyzed here. Laminar layers were identified functionally using visual evoked potential criteria. Specifically, layer 4 (granular) was identified by the largest current sink accompanying the polarity inversion of the N95 component of the visual evoked potential (Givre et al., 1994; Schroeder et al., 1998).

Anatomically a canonical circuit has been established in a cortical column (Lund, 2002; Douglas and Martin, 2004) in which axons from granular layer (G) cells synapse onto pyramidal cells in the supragranular layers (SG), which in turn synapse onto infragranular (IG) pyramidal neurons, and IG pyramidal neurons complete the circuit by sending axons into the G layers. Physiologically, alpha current generators have been identified in SG, G and IG layers of V4 (Bollimunta et al., 2008). Although largest alpha power is often observed in the SG layers (Haegens et al., 2015), using a variety of *in vitro* and *in vivo* techniques, past work has demonstrated that the primary alpha pacemaker is located in the IG layers (Lopes da Silva, 1991; Silva et al., 1991; Flint and Connors, 1996; Bollimunta et al., 2008), a finding that is also well-supported by biophysical models of neuronal oscillations (Carracedo et al., 2013). These considerations provide the “ground truth” for comparing the performance of various types of signals. Unipolar LFPs were taken from the contacts overlaying the alpha generators established by the PRAT method described above in the granular (G, contact 8) and infragranular (IG, contact 11) layers. For the bipolar derivations, the contacts used were: G = LFP(contact 9) − LFP(contact 7) and IG = LFP(contact 12) − LFP(contact 10), as shown in Figure 3A. Average re-referenced signals and CSD signals were derived as described above. The ground truth prediction is that IG → G is expected to be large and significant whereas G → IG small and statistically insignificant.

#### ECoG recordings from human MTL and PFC

**Figure 4:**
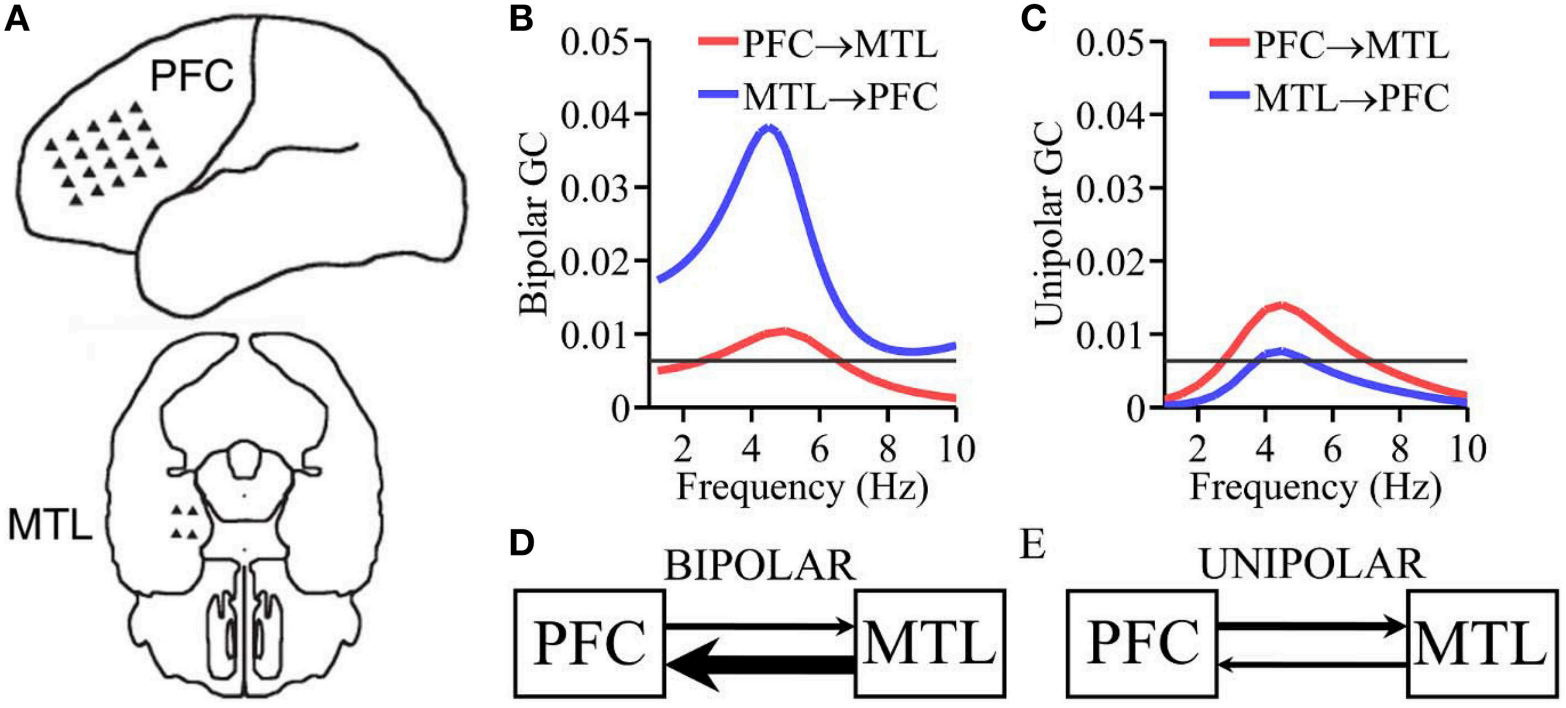
**ECoG recording from epilepsy patient. (A)** Placement of electrode arrays. **(B)** Granger causality spectra using bipolar data. **(C)** Granger causality spectra using unipolar data. **(D,E)** Schematic summarizing the Granger causality results where the thickness of the arrows is proportional to the corresponding Granger causality values.

Electrocorticogram (ECoG) data were recorded from implanted subdural electrodes in an epileptic patient. The patient gave informed consent and participated in the study. The experimental and recording protocol was approved by the Institutional Review Board of the University of Florida and the affiliated Shands Hospital at the University of Florida. Arrays of platinum–iridium electrodes embedded in silastic sheets (3 mm exposed diameter, 10 mm center-to-center spacing; Ad-tech Medical, Racine, WI, USA) were placed directly on the cortical surface. Figure 4A illustrates the approximate positions of the implanted electrode arrays. The electrode grid (20 electrodes) covered the left lateral prefrontal cortex (PFC) and the electrode strips (4 electrodes) covered the left medial temporal lobe (MTL). The reference electrode was an electrode fixed to the scalp of the subject. During resting state recording, the subject was instructed to visually fixate on the center of a computer screen, and minimize eye and body movement. Data were sampled at 400 Hz by a Nicolet amplifier system, band-pass filtered offline from 0.16 to 30 Hz, downsampled to 100 Hz, and divided into epochs of 640 ms in duration. A total of 321 epochs were analyzed here.

The pattern of causal interactions between PFC and MTL in humans is difficult to determine. DTI fiber tracking techniques cannot furnish directions of fiber projections (Audoin et al., 2007). However, anatomical studies in rodents have shown that the MTL structure hippocampus projects directly to PFC, whereas PFC projects back to the hippocampus indirectly through other brain structures such as the thalamus (Vertes et al., 2007; Cassel et al., 2013). Analogously, in rhesus monkeys, with the aid of anterograde neural tracers, studies have shown that the MTL structure amygdala projects directly to PFC, whereas PFC projects back indirectly through other structures such as orbitofrontal cortex (Rempel-Clower and Barbas, 2000; Barbas et al., 2005). These anatomical findings suggest that causal influence from MTL → PFC should be larger than PFC → MTL. This pattern has been found in a human ECoG study of memory retrieval where the causal influence between PFC and MTL was further shown to be mediated by theta oscillations (Anderson et al., 2010). The above considerations become the “ground truth” (albeit rather weak) for testing the performance of unipolar and bipolar signals. Bipolar derivations were obtained by taking the difference of unipolar signals in neighboring electrodes (vertical, horizontal, and diagonal) within each electrode array (PFC vs. MTL; Anderson et al., 2010), resulting in a total of 55 bipolar derivations for PFC and two bipolar derivations for MTL. For PFC-MTL Granger causality analysis was applied to all pairwise combinations of PFC signals and MTL signals (80 unipolar inter-areal pairs and 110 bipolar inter-areal pairs). Granger causality spectra were computed for each pairwise combination. Pairs with significant Granger causality (*p* < 0.05, see below) were then averaged (see below). Given the weakness of the basis used for making the prediction, this analysis should be considered exploratory, rather than confirmatory.

### Data analysis

#### Estimation of granger causality

Each pair of signals was subjected to autoregressive (AR) spectral analysis in which Granger causality spectral estimates were derived. The procedure has been described in detail in previous publications (Ding et al., 2000, 2006; Bollimunta et al., 2008). Each epoch was treated as the realization of an underlying stochastic process. The model order *m* was determined by a combination of the Akaike information criterion (Akaike, 1974) and further verification by minimizing mean square error between the spectral estimates from the AR model and that from the Fourier method. For each dataset analyzed in this study, the most appropriate *m* was chosen as a tradeoff between sufficient spectral resolution and over-parameterization.

#### Interpretation of granger causality

Statistically, for two simultaneously measured time series, one series can be called causal to the other if we can better predict the second series by incorporating past knowledge of the first one (Wiener, 1956). This concept was later adopted and formalized by Granger (1969) in the context of linear regression models of stochastic processes. Specifically, if the variance of the prediction error for the second time series at the present time is reduced by including past measurements from the first time series in the linear regression model, then the first time series can be said to have a causal (directional or driving) influence on the second time series. Reversing the roles of the two time series, one repeats the process to address the question of causal influence in the opposite direction. Here, in our simulation model as well as our three experimental preparations, directions of causal influence are equated with directions of synaptic transmission of neuronal activity (Ding et al., 2006; Bollimunta et al., 2008).

#### Testing of statistical significance

To test whether the estimated Granger causality in a given direction is greater than 0, we utilized a random permutation approach (Brovelli et al., 2005; Ding et al., 2006). In this approach a baseline null-hypothesis distribution is constructed from which statistical significance threshold can be derived. Specifically, for the two given time series, the epoch index from one was permuted randomly against that from the other to create a synthetic dataset, in which the temporal structure within each epoch is preserved but the interdependence between them destroyed. Granger causality spectra were derived from the synthetic dataset and the largest value was taken. This random permutation procedure was repeated five hundred times to yield the null-hypothesis distribution of Granger causality spectra. Granger causality values from the actual dataset were compared against the distribution and considered significant if they exceeded the 95th percentile value of the null hypothesis distribution (*p* < 0.05), plotted as black lines in Figures 1–4.

## Results

### Simulation model

As shown in Figure 1A, the model contained two brain areas, the XY area (red) and UV area (blue), with each composed of two interacting cortical columns. Each cortical column has an excitatory population and an inhibitory neuronal population that reciprocally interact. The full set of equations for the model was given in Methods. Unipolar signals are *x*_1_(*t*) − *R*(*t*), *x*_2_(*t*) − *R*(*t*), *u*_1_(*t*) − *R*(*t*), and *u*_2_(*t*) − *R*(*t*), and the bipolar signals representing XY area and UV area are *x*_1_(*t*) − *x*_2_(*t*) and *u*_1_(*t*) − *u*_2_(*t*), respectively. Here the common reference signal *R*(*t*) is a Gaussian white noise process.

Using bipolar signals, the Granger causality spectra in Figure 1B shows a unidirectional driving pattern, namely, XY → UY > 0 and UV → XY = 0, in agreement with the ground truth. In contrast, using unipolar signals, the Granger causality spectra in Figure 1C show a bidirectional driving pattern, namely, XY → UV > 0, UV → XY > 0, which is inconsistent with the ground truth. To examine the effect of the magnitude of the common signal *R*(*t*), we computed UV → XY using unipolar signals *x*_1_(*t*) − *R*(*t*) and *u*_1_(*t*) − *R*(*t*), and plotted it as a function of the ratio between the magnitude of *x*_1_(*t*) and that of *R*(*t*). As can be seen in Figure 1D, for sufficiently small *R(t)*, UV → XY falls below significance level, indicating that UV → XY become statistically indistinguishable from zero, namely, UV → XY = 0. This suggests that the correct network topology can be identified using unipolar signals only when the common reference activity is negligible.

### Laminar data from rat hippocampus

Based on the CSD profile of theta generators established by the PRAT method (Bollimunta et al., 2008) in Figure 2A, unipolar LFPs were taken from the channels overlaying the theta dipoles in DG hilus (contact 3) and CA1 (contact 13). For bipolar LFPs, the contacts used were: CA1 = LFP(contact 2) − LFP(contact 4) and DG = LFP(contact 12) − LFP(contact 14).

In Figure 2B, the bipolar LFP Granger causality spectra exhibit a clear peak at ~5 Hz in the DG → CA1 direction. The causality in the other direction, CA1 → DG, is below the significance threshold *p* = 0.05 (depicted as a black line in Figures 2B–E). This unidirectional Granger causality pattern, consistent with the ground truth, indicates that theta rhythm is directed from the largest dipole in DG to CA1 through the classic three-synaptic network of the hippocampus. In comparison, Figure 2C shows that Granger causality analysis using unipolar LFPs yielded bidirectional causal influence between DG and CA1, which is inconsistent with the ground truth. In Figure 2D, the Granger causality spectra based on CSD signals correctly detected the unidirectional causality in the DG → CA1 direction but failed to completely eliminate causal influence in the CA1 → DG direction. Figure 2E shows the Granger causality spectra derived from average re-referenced signals. Again the results showed causal influences both from CA1 → DG as well as in the opposing DG → CA1 direction. Figure 2F shows that the magnitude of the average reference signals from a typical epoch and that of the unipolar LFP from CA1 is comparable. The main results from the analysis of this dataset are schematically summarized in Figure 2G: unipolar and average re-referenced signals have resulted in connectivity patterns inconsistent with the anatomical prediction. Elimination of common signals by bipolar derivations allowed the detection of the correct causal influence directions predicted by anatomical connectivity. CSD treatment, which also eliminates common signals, yielded results that are close to the predicted connectivity pattern. The non-zero DG → CA1 component may be understood from the standpoint of noise amplification via differentiation (see Discussion Section).

### Laminar data from monkey V4

Based on the CSD profile of alpha generators established by the PRAT method (Bollimunta et al., 2008) shown in Figure 3A, unipolar LFPs were taken from channels overlaying the alpha generators in infragranular (IG, contact 11) and granular (G, contact 8) laminae. For bipolar LFPs, the contacts used were: IG = LFP(contact 10) − LFP(contact 12) and G = LFP(contact 7) − LFP(contact 9).

The bipolar LFP Granger causality spectrum of IG → G exhibits a clear peak at ~10 Hz (Figure 3B), whereas Granger causality in the other direction G → IG is not significant (*p* = 0.05 threshold line plotted in black), consistent with the ground truth. Figure 3C shows the Granger causality spectra using unipolar LFPs where a bi-directional causal influence pattern between IG and G layers was seen over a broad frequency range, which is not consistent with the ground truth. In Figure 3D, the Granger causality spectra based on CSD signals essentially identified the expected connectivity pattern, although the G → IG spectrum is at the threshold level, not quite under the threshold. Again, noise amplification that occurs during repeated differentiation may have played a role here (see Discussion Section). Figure 3E shows the Granger causality spectra derived from average re-referenced signals, which again shows a bi-directional causal influence pattern between IG and G layers over a broad frequency range, similar to the unipolar Granger causality spectra. Figure 3F shows that the average reference signal and the corresponding unipolar LFP from G layer have comparable magnitude. The main results from the analysis of this dataset are schematically summarized in Figure 3G: again, except for bipolar signals and somewhat less so for CSD signals, unipolar and average re-referenced signals resulted in connectivity patterns inconsistent with ground truth prediction. The presence of common signals is the main reason for the latter two types of signal treatments not being able to reveal the expected connectivity pattern.

### ECoG data from human PFC and MTL

ECoG data were recorded from implanted subdural electrodes covering the prefrontal cortex (PFC) and medial temporal lobe (MTL; Figure 4A) during fixation. In Figure 4B the Granger causality spectra calculated using the bipolar derivations shows a bidirectional flow of information, with the causal influence from MTL to PFC greater than the causal influence from PFC to MTL at the theta peak frequency ~5 Hz, namely, MTL → PFC > PFC → MTL. This pattern is consistent with what was found during memory recall (Anderson et al., 2010). Figure 4C shows the same functional relationship calculated using unipolar LFPs. In this case, the Granger causality spectra are also bidirectional, but with the causality from PFC → MTL greater than MTL → PFC. Figures 4D,E schematically summarizes the results.

## Discussion

Multielectrode signals are the basis for assessing the patterns of functional connectivity between neuronal ensembles and between brain areas. Because such signals are recorded against a common reference electrode, and the common reference electrode is generally not electrically silent, it is important to study the impact of such common signals on functional connectivity measures. Past work has discussed and demonstrated the confounding effects of common reference and volume conduction on functional connectivity measures in human scalp EEG recordings (Nunez et al., 1997; Nolte et al., 2004). Yet, in invasive recordings, this problem has received insufficient attention, possibly due to the fact that the electrodes are closer to the tissue being recorded from and as such it is therefore thought to be immune from the problem. Moreover, a series of reports estimate that the spatial spread of the LFP was very confined, on the order of a few hundred microns (Xing et al., 2005; Katzner et al., 2009), although the volume conduction model used in these reports has been questioned (Kajikawa and Schroeder, 2011, 2015). In light of these and earlier findings (Bollimunta et al., 2009), the present study aimed to examine the adverse effects of common signals on Granger causality estimation and propose to overcome these adverse effects by using bipolar derivations. To accomplish this we analyzed four datasets: a simulation model where connectivity pattern is known *a priori* and three neuroscientific electrophysiological examples where some degree of ground truth can be gained from past studies.

### Simulation model

Simulation is an essential tool for testing methods because the exact answer (ground truth) is known *a priori*. We applied the simulation technique to address the effects of common signal on Granger causality estimation. Interacting brain areas with built-in causality patterns were simulated by coupled non-linear differential equations to yield data that mimicked local field potential recordings. We used a simulation model (Kamiński et al., 2001) comprised of two interacting brain areas, denoted XY area and UV area, with each area comprised of two coupled cortical columns, where each column contained excitatory and inhibitory populations that interact with one another. The model parameters are chosen in such a way that the XY area unidirectionally drive the UV area. The results clearly showed that the network connectivity is correctly identified using bipolar signals. However, using unipolar signals, UV → XY is greater than zero, indicating that the causal connectivity is incorrectly identified. To further elucidate the adverse influence of the common signal, we used unipolar signals to examine the dependence of UV → XY on the magnitude of the common signal, and found that correct connectivity pattern can be identified only when the magnitude of the common signal is negligible.

### Laminar data from rat hippocampus

Testing methods on experimental data is not always straightforward. The main reason is that the ground truth is often unknown. This makes it difficult to differentiate methods based on their performance. In this study we have identified three examples where some degree of ground truth can be derived from the literature. The first example concerns theta (4–8 Hz) oscillations elicited by brainstem stimulation in a rat under urethane anesthesia where hippocampal LFPs were recorded via a multicontact linear electrode. In the classic three-synaptic circuit of the hippocampus, DG neurons synapse on pyramidal cells in CA3 via mossy fibers, which in turn synapse on CA1 pyramidal cells via Schaffer collaterals, resulting in an anatomically unidirectional DG → CA3 → CA1 pathway (Andersen et al., 1979; Amaral and Witter, 1989; Amaral et al., 2007). This predicts that the theta generator in DG should exert a significant unidirectional influence on the CA1 theta generator whereas the causal influence in the opposite direction is small and may be negligible. As expected, bipolar derivations yielded a unidirectional information flow from DG to CA1, consistent with the ground truth, whereas unipolar signals resulted in a bidirectional causal influence with a significant CA1 → DG, which is inconsistent with the anatomical prediction. We also pursued other signal treatments. Average re-referencing again did not yield the correct connectivity pattern. The CSD treatment, which eliminates common signals, correctly detected the causality in the DG → CA1 but did not completely remove the causal effect in the CA1 → DG direction, potentially due to noise amplification that occurs through repeated differentiation (see below). This example clearly supports our hypotheses that common signals adversely impact Granger causality and that the problem can be overcome by using bipolar derivations.

### Laminar data from monkey V4

Our second experimental example concerns alpha oscillations (8–12 Hz) in V4 in awake-behaving macaque where LFPs were recorded via a multicontact linear electrode. Anatomically, axons from granular layer cells synapse onto pyramidal cells in the supragranular layers. These neurons in turn send axons that synapse onto infragranular pyramidal neurons. Infragranular pyramidal neurons send axons into the granular and upper layers and complete the circuit. This is known as the canonical circuit (Douglas and Martin, 2004). Physiologically, early work on the genesis of the cortical alpha rhythm proposed that layer five pyramidal neurons are the pacemaker cells (Lopes da Silva, 1991). This hypothesis was supported by *in vitro* studies on isolated slices of rat sensorimotor neocortex where layer five pyramidal neurons were found necessary and sufficient to produce synchronized oscillations of 5–12 Hz (Silva et al., 1991) and by studies in awake-behaving macaques where the alpha generator in the infragranular layers acts as primary pacemaker of alpha (Bollimunta et al., 2008). Anatomical and physiological evidence thus converge to predict that the alpha generator in IG layers should exert a significant unidirectional influence on the G layer alpha generator whereas the causal influence in the opposite direction is small and may be negligible. The bipolar Granger causality is consistent with the ground truth, whereas unipolar signals yielded bidirectional causal influence between IG and G layers, which is not consistent with the ground truth. Average re-referencing again did not help to overcome the problem. The CSD treatment, which also eliminates common signals, essentially identified the correct pattern of interaction, but the G → IG magnitude is at or slightly above the threshold level, depending on frequency. This may again be understood through noise amplification via differentiation (see below).

### ECoG data from human subject

Functional MRI studies show that PFC and MTL functionally interact during memory-related cognitive processing (Grady et al., 2003; Gazzaley et al., 2004; Nee and Jonides, 2008). Anderson et al. showed that the interaction between MTL and PFC is mediated by theta oscillatory synchrony (Anderson et al., 2010). Whereas ground truth regarding the pattern of the MTL-PFC causal interactions is difficult to ascertain in humans, in rodents and non-human primates, it is known that MTL structures project to PFC directly, whereas PFC projection back to the MTL structures is indirect, mediated by other brain areas (Rempel-Clower and Barbas, 2000; Barbas et al., 2005; Vertes et al., 2007; Cassel et al., 2013). This might suggest that MTL → PFC is larger than PFC → MTL in the theta frequency band. A previous human ECoG study found this pattern during memory retrieval (Anderson et al., 2010). Analyzing intracranial ECoG data from an epilepsy patient undergoing presurgical evaluation for surgical therapy, we examined Granger causality between MTL and PFC during fixation, using both unipolar and bipolar signals. Connectivity patterns calculated using bipolar derivations agree with the expectation whereas that using unipolar signals contradict the expectation. It is worth noting however, that because of the lack of strong ground truth in this example, the results should be seen as exploratory rather than confirmatory.

### Average reference

In addition to the comparison between unipolar and bipolar signals, we have also considered the average re-referencing method. In this method data is averaged across all channels and this average is subtracted from each channel. Average re-referencing is widely used in human scalp EEG studies. For laminar recordings, theoretical work shows that voltage averaged across the entire cortical column should be zero (Nicholson and Freeman, 1975). Recent works have implemented average referencing in invasive recordings (Pohlmeyer et al., 2014; Steinmetz and Moore, 2014). Furthermore, Ludwig et al. (2009) note the common use of average referencing in scalp EEG and propose to use common average referencing in microelectrode arrays to generate a more ideal reference. In terms of overcoming the common signal problem, the average re-referencing method will work well if the average produces near-zero activities. However, this is often not the case, and previous studies have cautioned that an average reference may also produce erroneous connectivity estimates (Nunez et al., 1997). Here, for both the rat laminar data and monkey laminar data, average re-referencing yielded erroneous Granger causality results. Further examination demonstrates that the average reference signal is not close to zero and its magnitude is comparable to data from individual unipolar recording channels.

### Current source density

CSD involves the second spatial derivative of the laminar LFPs and is an index of cross-membrane current flow. Taking the second spatial derivative removes the common signal but at the same time amplifies noise. To see the reason assume that noise ξ_A_ and ξ_B_ from electrodes A and B are independent. The noise in the bipolar signal derived from A and B is ξ_A_ − ξ_B_ whose variance is equal to $\sigma_{\xi \text{A}}^{2}$ + $\sigma_{\xi \text{B}}^{2}$. In other words, the noise level is amplified through subtraction (first derivative). Three different LFP channels, A, B and C, are required to compute CSD. The noise of the second spatial derivative A + C − 2B has a variance of $\sigma_{\xi \text{A}}^{2}$ + $\sigma_{\xi \text{C}}^{2}$ + 4$\sigma_{\xi \text{B}}^{2}$ which represents further amplification of noise level. It has been shown that enhanced noise can cause spurious effects when computing Granger causality (Nalatore et al., 2007). In particular, the direction with zero Granger causality can become significant under the influence of noise. This may underlie the observed CSD-derived Granger causality patterns in both rat and monkey data. In both cases, although the expected connectivity patterns are close to being correctly identified, CA1 → DG in the rat and G → IG in the monkey showed Granger causality above or at the significance threshold, inconsistent with ground truth prediction and the bipolar results. The reason is likely noise amplification through differentiation. Future work may consider combining CSD-derived Granger causality with denoising approaches (Nalatore et al., 2007, 2009).

### Additional remarks

First, methods based on independent component analysis (ICA) have been proposed to localize neural signals. Korovaichuk et al. have suggested the combined use of ICA and CSD as high resolution methods to identify LFP components in Schaffer and perforant pathways and to quantify ongoing activity in selective electrical stimulation of known rat hippocampal pathways (Korovaichuk et al., 2010). Nolte and colleagues have suggested that ICA can be used as a method to test for artifacts of volume conduction prior to applying functional connectivity analyses (Shahbazi et al., 2010). However, there are also arguments against the use of PCA and ICA, citing that assumptions of orthogonality in such methods as invalid in the context of interacting neuronal populations (Gratiy et al., 2011). This concern is particularly relevant for the laminar data considered in this study as the neurons within the recorded compact structures are expected to be highly interactive. Despite such concerns, combining ICA and other source localization methods with Granger causality, especially in large-scale networks, is a promising approach and expected to be a fruitful area of exploration in the future.

Second, although the bipolar solution proposed here to deal with the common signal problem may impact experimental design, especially electrode placement, we are by no means proposing the use of bipolar montage for data acquisition, as this may lead to the irreversible loss of information. Rather, what we proposed is to use bipolar derivation as a *post hoc* analysis step, prior to computing functional connectivity measures such as Granger causality.

Third, bipolar derivation is shown to be readily applicable in many recording setups and makes minimum amount of assumptions. How to choose the two electrodes depends on the problem. For both the rat example and the monkey example, to avoid subtracting out signals, we did not use adjacent electrodes for bipolar derivations. However, the two electrodes cannot be too far apart so as to sample activities from different generators. The CSD profiles provide a useful guide for making that decision. Even with precaution there might be significant signal loss especially in the low frequency bands as low frequency activities may be more widely shared among electrodes. For the ECoG example this is not a major concern as the electrodes are sufficiently far apart and the question asked concerns large-scale brain structures.

Fourth, how common signals impact Granger causality estimation is a complex problem. Different kinds of signals may impact GC differently. An analytical treatment of this problem is not yet available. Some insight may be gained from the studies investigating the impact of measurement noise on Granger causality (Nalatore et al., 2007) since common signals are additive similar to measurement noise. Note that this situation is different from the common input problem which occurs at the level of neuronal processing. When a common input is introduced to the noise terms of a multivariate autoregressive process, and participates in the driving of the dynamics, we would expect that the causal relations among the variables are not substantially changed, because the common input mainly impacts the so-called instantaneous causality which is a separate quantity different from the commonly used directional Granger causality (Ding et al., 2006; Rajagovindan and Ding, 2008).

## Conclusion

Common reference and volume conduction introduce common signals in all recording electrodes. These unipolar signals may create a confounding influence on such connectivity measures as Granger causality and leads to erroneous results. Bipolar derivations are proposed here as a way to overcome the problem. For both simulation and experiments, the results show that bipolar derivations yield interpretable connectivity patterns, supported by independent lines of evidence, whereas unipolar signals do not. Average re-referencing magnifies uncertainties along several dimensions and does not help to overcome the problem. Second-derivative (CSD) treatment, where applicable, eliminates common signals but amplifies noise and related uncertainties in interpretation. However, it is possible that CSD when combined with denoising approaches may recover the interpretable causal relations.

## Author contributions

AT and MD designed research; AT, BN, and DK analyzed data; AT, BN, BK, CS, and MD wrote the paper.

### Conflict of interest statement

The authors declare that the research was conducted in the absence of any commercial or financial relationships that could be construed as a potential conflict of interest.
